# New combinatorial formulae for nested Bethe vectors II

**DOI:** 10.1007/s11005-025-01896-2

**Published:** 2025-01-28

**Authors:** Maksim Kosmakov, Vitaly Tarasov

**Affiliations:** 1 Department of Mathematical Sciences, University of Cincinnati, P.O. Box 210025, Cincinnati, OH 45221 USA; 2 Department of Mathematical Sciences, Indiana University Indianapolis, 402 North Blackford St, Indianapolis, IN 46202-3216 USA

**Keywords:** Weight functions, Nested Bethe vectors, Algebraic Bethe ansatz

## Abstract

We give new combinatorial formulae for vector-valued weight functions (off-shell nested Bethe vectors) for the evaluation modules over the Yangian $Y(\mathfrak {gl}_n)$.

## Introduction

In this paper, we construct new combinatorial formulae for vector-valued weight functions for evaluation modules over the Yangian $Y(\mathfrak {gl}_n)$. The weight functions, otherwise called (off-shell) nested Bethe vectors, are quite important in the theory of quantum integrable models and representation theory of Lie algebras and quantum groups. Their initial definition originates in the framework of the nested algebraic Bethe ansatz, an ingenious technique to find eigenvectors and eigenvalues of transfer matrices of lattice integrable models associated with higher-rank Lie algebras [4, 5]. An excellent review of the algebraic Bethe ansatz can be found in [18, 19]. The results of [5] have been extended to higher transfer matrices in [10].

On the other hand, the vector-valued weight functions are an essential part of construction of hypergeometric solutions to the quantized (difference) Knizhnik–Zamolodchikov equations [9, 23]. They also appeared in various related problems [3, 11, 24, 25]. In the last decade, the weight functions were connected to the stable envelopes for cotangent bundles of partial flag varieties that are particular examples of Nakajima quiver varieties, see [14–17, 26].

For various problems, it is desirable to have sufficiently explicit expressions for vector-valued weight functions for tensor products of evaluation modules over $Y(\mathfrak {gl}_n)$. Such expressions can be constructed in two steps. The first one is to deal with a single evaluation module and the second one is to merge expressions for individual evaluation modules into an expression for the whole tensor product. In this paper, we will consider the first step of this construction. The second step is fairly standard, see Theorem 3.5 in [22].

An evaluation $Y(\mathfrak {gl}_n)$-module is the pull-back of a $\mathfrak {gl}_n$-module via the evaluation homomorphism $Y(\mathfrak {gl}_n)\rightarrow U(\mathfrak {gl}_n)$, see Sect. 2. The goal in general is to expand the vector-valued weight function for the evaluation $Y(\mathfrak {gl}_n)$-module in some natural basis of the underlying $\mathfrak {gl}_n$-module and find expressions for the coordinates. For Verma modules over $\mathfrak {gl}_n$, such kind of expressions were written in [22]. In this paper, we give a generalization of formulae from [22].

Combinatorial formulae for the vector-valued weight functions associated with the differential Knizhnik–Zamolodchikov equations were developed in [1, 7, 8, 13, 20, 21].

The expressions for weight functions in [22] are based on recursions induced by the standard embeddings of Lie algebras, $\,\mathfrak {gl}_1\oplus \mathfrak {gl}_{n-1}\subset \mathfrak {gl}_n\,$ and $\;\mathfrak {gl}_{n-1}\oplus \mathfrak {gl}_1 \subset \mathfrak {gl}_n$. The recursions allow one to write down weight functions for $Y(\mathfrak {gl}_n)$ via weight functions for $Y(\mathfrak {gl}_{n-1})$. This results in formulae for coordinates of weight functions in bases of Verma $\mathfrak {gl}_n$-modules of the form

1.1 $$\begin{aligned} \Bigl \{ \prod _{i>j} e_{ij}^{m_{ij}}v, \quad m_{ij}\in \mathbb {Z}_{\geqslant 0}\Bigr \}, \end{aligned}$$

where $e_{ij}$ are the standard generators of $\mathfrak {gl}_n$, $\;v$ is the highest weight vector, and a certain ordering of noncommuting factors $e_{ij}^{m_{ij}}$ in the product is imposed. The ordering is determined by a chain of embeddings $\;\mathfrak {gl}_1 \oplus \dots \oplus \mathfrak {gl}_1\subset \dots \subset \mathfrak {gl}_n$, where the *k*-th element of the chain is induced either by the embedding $\,\mathfrak {gl}_1\oplus \mathfrak {gl}_{k-1}\subset \mathfrak {gl}_k\,$ or by the embedding $\;\mathfrak {gl}_{k-1}\oplus \mathfrak {gl}_1 \subset \mathfrak {gl}_k$. For instance, the chain

$$\begin{aligned} \mathfrak {gl}_1 \oplus \dots \oplus \mathfrak {gl}_1\subset \mathfrak {gl}_2 \oplus \mathfrak {gl}_1 \oplus \dots \oplus \mathfrak {gl}_1\subset \dots \subset \mathfrak {gl}_{n-2}\oplus \mathfrak {gl}_1 \oplus \mathfrak {gl}_1 \subset \mathfrak {gl}_{n-1}\oplus \mathfrak {gl}_1 \subset \mathfrak {gl}_n \end{aligned}$$

yields the orderings

$$\begin{aligned} e_{ij}^{m_{ij}} \,{\text { is to the left of }}\, e_{kl}^{m_{kl}} \,{\text { if }}\, i>k \,{\text {or}}\, i=k, j>l, \end{aligned}$$

while the chain

$$\begin{aligned} \mathfrak {gl}_1 \oplus \dots \oplus \mathfrak {gl}_1\subset \mathfrak {gl}_1 \oplus \dots \oplus \mathfrak {gl}_1\oplus \mathfrak {gl}_2\subset \dots \subset \mathfrak {gl}_1 \oplus \mathfrak {gl}_1\oplus \mathfrak {gl}_{n-2} \subset \mathfrak {gl}_1\oplus \mathfrak {gl}_{n-1}\subset \mathfrak {gl}_n \end{aligned}$$

yields the orderings

$$\begin{aligned} e_{ij}^{m_{ij}} \,{\text { is to the left of }}\, e_{kl}^{m_{kl}} \,{\text { if }}\, j<l \,{\text { or }}\, j=l, i<k. \end{aligned}$$

These two orderings are examples of normal orderings of the factors $e_{ij}^{m_{ij}}$ corresponding to normal orderings of positive roots of the Lie algebra $\,\mathfrak {gl}_n$. An ordering is called normal is for any $i>j>k$, the factor $e_{ik}^{m_{ik}}$ is located between the factors $e_{ij}^{m_{ij}}$ and $e_{jk}^{m_{jk}}$.

Formulae established in [22] do not cover all normal orderings of noncommuting factors in (1.1). The first example occurs at $n=4$ and looks as follows,

1.2 $$\begin{aligned} \Bigl \{e_{32}^{m_{32}}e_{31}^{m_{31}}e_{42}^{m_{42}}e_{41}^{m_{41}} e_{21}^{m_{21}}e_{43}^{m_{43}}v,\qquad m_{ij}\in \mathbb {Z}_{\geqslant 0}\Bigr \}. \end{aligned}$$

This example and similar ones for $n>4$ are important in applications, see for instance [8].

To enlarge the set of covered orderings, we consider recursions based on embeddings

$$\begin{aligned} \mathfrak {gl}_m\oplus \mathfrak {gl}_{n-m}\subset \mathfrak {gl}_n \qquad {\text {with}}\, \;\; 1<m<n-1. \end{aligned}$$

For instance, the embedding $\mathfrak {gl}_2\oplus \mathfrak {gl}_2\subset \mathfrak {gl}_4$ yields example (1.2). We worked out example (1.2) in detail in our previous paper [6]. In this paper, we consider the general case. The reasoning is very similar to the $\,\mathfrak {gl}_4$ case, and we suggest taking a look into [6] to clarify technicalities if needed.

The main result of the paper is Theorem 5.5. It gives an expression for the weight function for $Y(\mathfrak {gl}_n)$ via the weight functions for $Y(\mathfrak {gl}_m)$ and $Y(\mathfrak {gl}_{n-m})$. For $m=1$ and $m=n-1$, Theorem 5.5 becomes, respectively, Theorem 3.3 and Theorem 3.1 in [23].

Unlike [22], we will consider only the case of weight functions for Yangian modules (the rational case). The weight functions for modules over the quantum loop algebra $U_q(\widetilde{\mathfrak {gl}_n})$, the trigonometric case, cannot be dealt with by our current approach because of essential noncommutativity of *q*-analogues of the generators $e_{ij}$, $\;i>j$. For instance, the obstacle in example (1.2) comes from the relation

$$\begin{aligned} e_{42}e_{31}-e_{31}e_{42}=(q-q^{-1})e_{32}e_{41} \end{aligned}$$

that holds in the trigonometric case. This kind of obstruction does not show up in [22], but prevents one from extending the reasoning used in this paper to the trigonometric case.

An alternative approach to get explicit expressions for the vector-valued weight functions in the trigonometric case was developed in [2, 3, 12]. It is based on considering composed currents and half-currents in the quantum affine algebra and their projections on two Borel subalgebras of different kinds. Although currents and half-currents are rather complicated expressions in the original generators of the quantum affine algebra, their commutation relations turn out to have a simple form in certain cases. This approach allows one to recover combinatorial expression for vector-valued weight functions in evaluation modules in the trigonometric case obtained in [22]. It is an interesting question to obtain trigonometric analogues of new combinatorial expressions for vector-valued weight functions developed in this paper within the composed currents approach. This might require working out commutation relations of composed currents with interlacing indices, like $F_{3,1}(t)$ and $F_{4,2}(t)$ in the notation of [3].

## Notations

We will be using the standard superscript notation for embeddings of tensor factors into tensor products. For a tensor product of vector spaces $V_1\otimes V_2\otimes \cdots \otimes V_k$ and an operator $A\in {{\,\textrm{End}\,}}(V_i)$, denote

$$\begin{aligned} A^{(i)}=1^{\otimes (i-1)} \otimes A \otimes 1^{\otimes (k-i)} \in {{\,\textrm{End}\,}}(V_1\otimes V_2\otimes \cdots \otimes V_k). \end{aligned}$$

Also, if $B\in {{\,\textrm{End}\,}}(V_j)$, $i\ne j$, denote $(A\otimes B)^{(ij)}=A^{(i)}B^{(j)}$, etc.

Fix a positive integer *n*. All over the paper we identify elements of End $\mathbb {C}^n$ with $n\times n$ matrices using the standard basis of $\mathbb {C}^n$. That is, for $L\in {{\,\textrm{End}\,}}\mathbb {C}^n$ we have $\ L=\left( L_b^a \right) _{a,b=1}^n$, where $L_b^a$ are the entries of *L*. Entries of matrices acting in the tensor products $\left( \mathbb {C}^n\right) ^{\otimes k}$ are naturally labeled by multi-indices. For instance, if $M\in {{\,\textrm{End}\,}}\left( \mathbb {C}^n\otimes \mathbb {C}^n\right)$, then $M=\left( M_{cd}^{ab} \right) _{a,b,c,d=1}^n$.

The rational *R*-matrix is $R(u)\in {{\,\textrm{End}\,}}(\mathbb {C}^n\otimes \mathbb {C}^n)$,

2.1 $$\begin{aligned} R(u)=1+\dfrac{1}{u}\sum _{a, b=1}^{n} E_{a b} \otimes E_{b a}, \end{aligned}$$

where $E_{a b} \in {{\,\textrm{End}\,}}\left( \mathbb {C}^{n}\right)$ is the matrix with the only nonzero entry equal to 1 at the intersection of the *a*-th row and *b*-th column. The entries of *R*(*u*) are

2.2 $$\begin{aligned} R^{ab}_{cd}(u)= \delta _{ac}\delta _{bd} +\dfrac{1}{u}\delta _{ad}\delta _{bc}. \end{aligned}$$

The *R*-matrix satisfies the Yang–Baxter equation

$$\begin{aligned} R^{(12)}(u-v) R^{(13)}(u) R^{(23)}(v)=R^{(23)}(v) R^{(13)}(u) R^{(12)}(u-v). \end{aligned}$$

The Yangian $Y(\mathfrak {g l}_{n})$ is a unital associative algebra with generators $\left( T^a_b\right) ^{\{s\}}, a, b=1, \ldots , n,$ and $s=1,2, \ldots$. Organize them into generating series:

2.3 $$\begin{aligned} T^a_b(u)=\delta _{ab}+\sum _{s=1}^{\infty } \left( T^a_b\right) ^{\{s\}} u^{-s}, \quad a, b=1, \ldots , n .\end{aligned}$$

The defining relations in $Y(\mathfrak {g l}_{n})$ are

2.4 $$\begin{aligned} (u-v)\left[ T^a_b(u), T^c_d(v)\right] =T^a_d(u)T^c_b(v) -T^a_d(v)T^c_b(u) \end{aligned}$$

for all $$a, b, c, d=1, \ldots , n.$$

Combine series (2.3) into a matrix $T(u)=\sum \nolimits _{a, b=1}^{n} E_{a b} \otimes T_{ b}^a(u)$ with entries in $Y(\mathfrak {g l}_{n})$. Then, relations (2.4) amount to the following equality

$$\begin{aligned} R^{(12)}(u-v) T^{(1)}(u) T^{(2)}(v)=T^{(2)}(v) T^{(1)}(u) R^{(12)}(u-v) , \end{aligned}$$

where $$T^{(1)}(u)=\sum \nolimits _{a, b=1}^{n} E_{a b}\otimes 1 \otimes T_{ b}^a(u)$$ and $$T^{(2)}(v)=\sum \nolimits _{a, b=1}^{n} 1 \otimes E_{a b}\otimes T_{ b}^a(v)$$.

The Yangian $Y(\mathfrak {g l}_{n})$ is a Hopf algebra. In terms of generating series (2.3), the coproduct $\Delta : Y(\mathfrak {g l}_{n}) \rightarrow Y(\mathfrak {g l}_{n}) \otimes Y(\mathfrak {g l}_{n})$ reads as follows:

2.5 $$\begin{aligned} \Delta \left( T_{ b}^a(u)\right) =\sum _{c=1}^{n} T^c_{ b}(u) \otimes T^a_{ c}(u), \quad a, b=1, \ldots , n . \end{aligned}$$

Denote by $\widetilde{\Delta }:Y(\mathfrak {g l}_{n}) \rightarrow Y(\mathfrak {g l}_{n}) \otimes Y(\mathfrak {g l}_{n})$ the opposite coproduct

2.6 $$\begin{aligned} \widetilde{\Delta }\left( T_{ b}^a(u)\right) =\sum _{c=1}^nT^a_c(u)\otimes T^c_b(u), \quad a, b=1, \ldots , n . \end{aligned}$$

Fix a collection of nonnegative integers $\xi _1,\xi _2,\ldots ,\xi _{n-1}$. Set $\varvec{\xi }=(\xi _1,\xi _2,\ldots ,\xi _{n-1})$ and $\xi ^a=\xi _1+\cdots +\xi _a$, $a=1,\ldots ,n-1$. Consider the variables $t^a_i$, $a=1,\ldots , n-1$, $i=1,\ldots \xi _a$. We will write

2.7 $$\begin{aligned} \varvec{t}^a=(t^a_1,\ldots , t^a_{\xi _a}),\qquad \varvec{t}=(\varvec{t}^1,\ldots ,\varvec{t}^{n-1}). \end{aligned}$$

We will use the ordered product notation for any noncommuting factors $X_1,\ldots ,X_k$,

$$\begin{aligned} \overset{\rightarrow }{\prod \limits _{1\leqslant i\leqslant k}} X_i= X_1X_2\ldots X_k, \quad \quad \overset{\leftarrow }{\prod \limits _{1\leqslant i\leqslant k}} X_i= X_kX_{k-1}\ldots X_1. \end{aligned}$$

Consider the vector space $(\mathbb {C}^n)^{\otimes \xi ^{n-1}}$ and define

$$\begin{aligned} \overset{[j]}{\mathbb {T}}({\varvec{t^j}})=\overset{\rightarrow }{\prod \limits _{ 1\leqslant k\leqslant \xi _j}} {T}^{(\xi ^{j-1}+k)}(t_k^j),\quad \quad \overset{[k,j]}{\mathbb {R}}(\varvec{t^k},\varvec{t^j})\\ = \overset{\rightarrow }{\prod \limits _{1\leqslant i\leqslant \xi _k}}\Biggl (\; \overset{\leftarrow }{\prod \limits _{1\leqslant l\leqslant \xi _j}} {R}^{(\xi ^{k-1}+i,\xi ^{j-1}+l)}(t^k_i-t^j_l)\Biggr ), \end{aligned}$$

where we view ${T}^{(\xi ^{j-1}+k)}(t_k^j)$ as a matrix with entries in $Y(\mathfrak {gl}_{n})$ acting on $(\xi ^{j-1}+k)$-th copy of $\mathbb {C}^n$ in $(\mathbb {C}^n)^{\otimes \xi ^{n-1}}$. For the expression

2.8 $$\begin{aligned} \widehat{\mathbb {T}}_{\varvec{\xi }}(\varvec{t})=\ \overset{[1]}{\mathbb {T}}(\varvec{t}^1)\ldots \overset{[n-1]}{\mathbb {T}}(\varvec{t}^{n-1})\overset{\leftarrow }{\prod \limits _{1\leqslant i\leqslant n-1}}\Biggl (\;\overset{\leftarrow }{\prod \limits _{1\leqslant j< i}}\overset{[i,j]}{\mathbb {R}}(\varvec{t^i},\varvec{t^j})\Biggr ), \end{aligned}$$

denote by ${\mathbb {B}}_{\varvec{\xi }}(\varvec{t})$, the following entry

2.9 $$\begin{aligned} {\mathbb {B}}_{\varvec{\xi }}(\varvec{t})=\left( \widehat{\mathbb {T}}_{\varvec{\xi }}(\varvec{t}) \right) ^{\varvec{1}^{\xi _1}, \,\varvec{2}^{\xi _2},\,\ldots ,\,\varvec{n-1}^{\xi _{n-1}}}_{\varvec{2}^{\xi _1},\,\varvec{3}^{\xi _2},\,\ldots ,\,\varvec{n}^{\xi _{n-1}}}, \end{aligned}$$

where

$$\begin{aligned} \begin{aligned}&\varvec{1}^{\xi _1}, \varvec{2}^{\xi _2}, \ldots , \varvec{(n-1)}^{\xi _{n-1}} =\underbrace{ 1, 1,\ldots 1}_{\xi _1},\;\underbrace{ 2,2,\ldots 2}_{\xi _2},\;\ldots \;,\underbrace{n-1,\;n-1,\;\ldots ,\;n-1}_{\xi _{n-1}}\;,\\&\varvec{2}^{\xi _1}, \varvec{3}^{\xi _2}, \ldots , \varvec{n}^{\xi _{n-1}} =\underbrace{ 2, 2,\ldots , 2}_{\xi _1},\;\underbrace{ 3,3,\ldots ,3}_{\xi _2},\;\ldots , \;\underbrace{n,\, n,\,\ldots ,\, n}_{\xi _{n-1}}\;. \end{aligned} \end{aligned}$$

To indicate the dependence on *n*, if necessary, we will write $\mathbb {B}_{\varvec{\xi }}^{\langle n\rangle }(\varvec{t})$.

There is a one-parameter family of automorphisms $\rho _x$: $Y(\mathfrak {gl}_n) \rightarrow Y(\mathfrak {gl}_n)$ defined in terms of the series *T*(*u*) by the rule $\rho _x T(u)= T(u-x)$, where in the right-hand side, each expression $(u-x)^{-s}$ has to be expanded as a power series in $u^{-1}$.

Denote, by $e_{ab}$, *a*,$b = 1,\ldots ,n$, the standard generators of the Lie algebra $\mathfrak {gl}_n$. A vector *v* in a $\mathfrak {gl}_n$ -module is called singular of weight $(\Lambda ^1,\ldots ,\Lambda ^n)$ if $e_{ab}v = 0$ for all $a<b$ and $e_{aa}v=\Lambda _av$ for all $a=1,\ldots ,n$.

The Yangian $Y(\mathfrak {g l}_{n})$ contains the universal enveloping algebra $U(\mathfrak {g l}_{n})$ as a Hopf subalgebra. The embedding is given by the rule $e_{a b} \mapsto (T_{ a}^b)^{\{1\}}$ for all $a, b=1, \ldots , n$. We identify $U(\mathfrak {g l}_{n})$ with its image in $Y(\mathfrak {g l}_{n})$ under this embedding.

The evaluation homomorphism $\epsilon$: $Y (\mathfrak {gl}_n) \rightarrow U(\mathfrak {gl}_n)$ is given by the rule $\epsilon$: $(T_{b}^a)(u) \mapsto \delta _{ab}+e_{ba}u^{-1}$ for all $a,b = 1,...,n$. Both the automorphisms $\rho _x$ and the homomorphism $\epsilon$ restricted to the subalgebra $U(\mathfrak {gl}_n)$ are the identity maps.

For a $\mathfrak {gl}_n$-module *V*, denote by *V*(*x*) the $Y(\mathfrak {gl}_n)$-module induced from *V* by the homomorphism $\epsilon \circ \rho _x$. The module *V*(*x*) is called an evaluation module over $Y(\mathfrak {gl}_n)$.

A vector *v* in a $Y(\mathfrak {gl}_n)$-module is called singular with respect to the action of $Y(\mathfrak {gl}_n)$ if $T^{a}_{b}(u)v = 0$ for all $1\leqslant b < a \leqslant n$. A singular vector *v* that is an eigenvector for the action of $T_{1}^{1}(u)$,...,$T_{n}^{n}(u)$ is called a weight singular vector, and the respective eigenvalues are denoted by $\langle T^1_{1}(u)v\rangle ,\ldots , \langle T^n_{n}(u)v\rangle$.

### Example

Let *V* be a $\mathfrak {g l}_{n}$-module and $v \in V$ be a $\mathfrak {gl}_n$-singular vector of weight $\left( \Lambda ^{1}, \ldots , \Lambda ^{n}\right)$. Then, *v* is a weight singular vector with respect to the action of  $Y(\mathfrak {g l}_{n})$ in the evaluation module *V*(*x*) and $\left\langle T^a_{ a}(u) v\right\rangle =1+\Lambda ^{a}(u-x)^{-1}$, $a=1, \ldots , n$.

For $k<n$, we consider two embeddings of the algebra $Y(\mathfrak {gl}_{k})$ into $Y(\mathfrak {gl}_{n})$, called $\phi _k$ and $\psi _k$:

2.10 $$\begin{aligned} \phi _k(T^{\langle k\rangle }(u))^a_b=(T^{\langle n\rangle }(u))^a_b \hspace{2cm} \psi _k(T^{\langle k\rangle }(u))^a_b=(T^{\langle n\rangle }(u))^{a+n-k}_{b+n-k}(u) \end{aligned}$$

$a,b=1,\ldots , k$. Here $(T^{\langle k\rangle }(u))^a_b$ and $((T^{\langle n\rangle }(u))^a_b$ are the series $T^a_b(u)$ for the algebras $Y(\mathfrak {gl}_{k})$ and $Y(\mathfrak {gl}_{n})$, respectively.

## Splitting property

Let $T_{a b}^{\langle r\rangle }(u)$ be series (2.3) for the algebra $Y(\mathfrak {g l}_{r})$, and $R^{\langle r\rangle }(u)$ be the corresponding rational *R*-matrix, see (2.1). For the rest of the paper, we fix integers *m* and *n*, $1\leqslant m<n$.

Consider $Y(\mathfrak {g l}_{n-m})$-module structure on the vector space $\mathbb {C}^{n-m}$ given by the rule

3.1 $$\begin{aligned} \pi (x): T^{\langle n-m \rangle }(u) \mapsto (u-x)^{-1} R^{\langle n-m\rangle }(u-x). \end{aligned}$$

Let $\mathrm {\textbf{v}}_{ 1}, \mathrm {\textbf{v}}_{ 2},\ldots , \mathrm {\textbf{v}}_{ n-m}$ be the standard basis of the space $\mathbb {C}^{n-m}$. The $Y(\mathfrak {g l}_{n-m})$-module defined by (3.1) is a highest weight evaluation module with $\mathfrak {g l}_{n-m}$ highest weight $(1,\ldots ,0,0)$ and highest weight vector $\mathrm {\textbf{v}}_{ 1}$.

Consider $Y(\mathfrak {g l}_{m})$-module structure on the vector space $\mathbb {C}^{m}$ given by the rule

3.2 $$\begin{aligned} \varpi (x): T^{\langle m\rangle }(u) \mapsto (x-u)^{-1}\Bigl (\Bigl (R^{\langle m\rangle }(x-u)\Bigr )^{(21)}\Bigr )^{t_{2}}, \end{aligned}$$

where the superscript $t_{2}$ stands for the matrix transposition in the second tensor factor.

We denote the standard basis of the space $\mathbb {C}^{m}$ by $\textbf{w}_{1}, \textbf{w}_{ 2},\ldots , \textbf{w}_{m}$. The $Y(\mathfrak {g l}_{m})$-module defined by (3.2) is a highest weight evaluation module with $\mathfrak {g l}_{m}$ highest weight $(0,\ldots ,0,-1)$ and highest weight vector $\textbf{w}_{m}$.

For any $Z\in$ $\operatorname {End}(\mathbb {C}^{n-m})$, set ${\nu }(Z)=Z \mathrm {\textbf{v}}_{ 1}$, and for any $X \in$ $\operatorname {End}(\mathbb {C}^{m})$, set $\bar{\nu }(X)=X \textbf{w}_{m}$. Recall the coproducts $\Delta$ and $\widetilde{\Delta }$, see (2.5) and (2.6), and the embeddings $\psi _{n-m}: Y(\mathfrak {g l}_{n-m})\rightarrow Y(\mathfrak {g l}_{n})$, $\phi _{m}: Y(\mathfrak {g l}_{m})\rightarrow Y(\mathfrak {g l}_{n})$ given by (2.10). For any *r*, denote by $(\Delta ^{\langle r\rangle })^{(k)}: Y(\mathfrak {g l}_{r}) \rightarrow \left( Y(\mathfrak {g l}_{r})\right) ^{\otimes (k+1)}$ and $(\widetilde{\Delta }^{\langle r\rangle })^{(k)}: Y(\mathfrak {g l}_{r}) \rightarrow \left( Y(\mathfrak {g l}_{r})\right) ^{\otimes (k+1)}$ the corresponding iterated coproduct and opposite coproduct. Consider the maps

$$\begin{aligned} \psi _{n-m}\left( x_{1}, \ldots , x_{k}\right) : Y(\mathfrak {g l}_{n-m}) \rightarrow \left( \mathbb {C}^{n-m}\right) ^{\otimes k} \otimes Y(\mathfrak {g l}_{n}),\end{aligned}$$

$$\begin{aligned} \psi _{n-m}(x_{1}, \ldots , x_{k})= ( {\nu }^{\otimes k} \otimes \textrm{id}) \circ \left( \pi \left( x_{1}\right) \otimes \cdots \otimes \pi \left( x_{k}\right) \otimes \psi _{n-m}\right) \circ (\Delta ^{\langle n-m\rangle })^{(k)}, \end{aligned}$$

and

$$\begin{aligned}\phi _{m}\left( x_{1}, \ldots , x_{k}\right) : Y(\mathfrak {g l}_{m}) \rightarrow \left( \mathbb {C}^{m}\right) ^{\otimes k} \otimes Y(\mathfrak {g l}_{n}) ,\end{aligned}$$

$$\begin{aligned} \phi _{m}\left( x_{ 1}, \ldots , x_{k}\right) = (\bar{\nu }^{\otimes k} \otimes \textrm{id}) \circ \left( \varpi \left( x_{ 1}\right) \otimes \cdots \otimes \varpi \left( x_{k}\right) \otimes \phi _{m} \right) \circ (\widetilde{\Delta }^{\langle m\rangle })^{(k)}. \end{aligned}$$

For any element $g \in \left( \mathbb {C}^{n-m}\right) ^{\otimes k} \otimes Y(\mathfrak {g l}_{n})$, we define its components $g^{\varvec{b}}$, $\varvec{b}=(b_{1}, \ldots , b_{k})$, by the rule

$$\begin{aligned} g=\sum _{b_{1}, \ldots , b_{k}=m+1}^{n} \mathrm {\textbf{v}}_{b_{1}-m} \otimes \cdots \otimes \mathrm {\textbf{v}}_{b_{k}-m} \otimes g^{\varvec{b}} . \end{aligned}$$

For any element $h \in \left( \mathbb {C}^{m}\right) ^{\otimes k} \otimes Y(\mathfrak {g l}_{n})$, we define its components $h^{\varvec{a}}$, $\varvec{a}=(a_{1}, \ldots , a_{k})$, by the rule

$$\begin{aligned} h=\sum _{a_{1}, \ldots , a_{k}=1}^{m} \textbf{w}_{a_{1}} \otimes \cdots \otimes \textbf{w}_{a_{k}} \otimes h^{\varvec{a}} . \end{aligned}$$

Given nonnegative integers $\xi _1,\ldots ,\xi _{n-1}$, and the variables $\varvec{t}=(t_1^1,\ldots , t^{n-1}_{\xi _{n-1}})$, see (2.7), denote

3.3 $$\begin{aligned} \begin{aligned}&\varvec{\xi }=(\xi _1,\ldots ,\xi _{n-1}),\\&\dot{\varvec{\xi }}=(\xi _1,\ldots ,\xi _{m-1}),\\&\ddot{\varvec{\xi }}=(\xi _{m+1},\ldots ,\xi _{n-1}),\\&\varvec{t}=(t^1_1,\ldots ,t^1_{\xi _1};t^2_1,\ldots ,t^2_{\xi _2};t^{n-1}_1,\ldots ,t^{n-1}_{\xi _n-1}),\\&\dot{\varvec{t}}=(t^1_1,\ldots ,t^1_{\xi _1};t^2_1,\ldots ,t^2_{\xi _2};t^{m-1}_1,\ldots ,t^{m-1}_{\xi _m-1}),\\&\ddot{\varvec{t}} =(t^{m+1}_1,\ldots ,t^{m+1}_{\xi _{m+1}};t^{m+2}_1,\ldots ,t^{m+2}_{\xi _{m+2}};t^{n-1}_1,\ldots ,t^{n-1}_{\xi _n-1}). \end{aligned} \end{aligned}$$

Recall that $$\xi ^a=\xi _1+\xi _2+\cdots +\xi _a$$, $$a=1,\ldots ,n-1$$.

Let *V* be a $\mathfrak {gl}_n$-module, $x\in \mathbb {C}$, and *V*(*x*) be the evaluation $Y(\mathfrak {g l}_{n})$-module. Consider a weight singular vector $v\in V(x)$ of $\mathfrak {gl}_n$-weight $\varvec{\Lambda }=(\Lambda _1,\ldots ,\Lambda _n)$. That is, we have

$$\begin{aligned} T^a_b(u)v=0,\; 1\leqslant b<a\leqslant n, \quad T_{a}^av=\dfrac{u-x+\Lambda _a}{u-x}v, \;a=1,\ldots ,n. \end{aligned}$$

We are interested in finding a formula for the vector $\mathbb {B}_{\varvec{\xi }}(\varvec{t})v$, where $\mathbb {B}_{\varvec{\xi }}(\varvec{t})$ is given by (2.9).

### Proposition 3.1

Let $v\in V(x)$ be a weight singular vector and $\,\varvec{\xi }=(\xi _1,\ldots ,\xi _{n-1})$ be a collection nonnegative integers. Then

3.4 $$\begin{aligned} \mathbb {B}_{\varvec{\xi }}(\varvec{t})v =\ \sum _{\varvec{a},\varvec{b}} {\mathcal {T}} (\varvec{t}^{m})^{\varvec{a}}_{\varvec{b}} \;\Bigl (\phi _m(\varvec{t}^m)\left( \mathbb {B}_{\dot{\xi } }^{\langle m\rangle }(\dot{\varvec{t}})\right) \Bigr )^{\varvec{a}} \Bigl (\psi _m(\varvec{t}^{m})\left( \mathbb {B}_ {\ddot{\xi }}^{\langle n-m\rangle }(\ddot{\varvec{t}})\right) \Bigr )^{\varvec{b}} v, \end{aligned}$$

where the sum is taken over all sequences $\varvec{a}=(a_1,a_2,\ldots ,a_{\xi _m})$, $\varvec{b}=(b_1,b_2,\ldots ,b_{\xi _m})$, such that $a_i\in \{1,2,\ldots , m\}$, $b_i\in \{m+1,m+2,\ldots ,n\}$ for all $i=1,\ldots ,\xi _m$, and

3.5 $$\begin{aligned} {\mathcal {T}}(\varvec{t}^m)^{\varvec{a}}_{\varvec{b}}=T(t^m_1)^{a_1}_{b_1}T(t^m_2)^{a_2}_{b_2}\ldots T(t^m_{\xi _m})^{a_{\xi _m}}_{b_{\xi _m}}\,. \end{aligned}$$

### Proof

Using the definition of the maps $\psi _m(\varvec{t}^{m})$ and $\phi _m(\varvec{t}^{m})$, formula (3.4) can be written as:

3.6 $$\begin{aligned} \begin{aligned}&\mathbb {B}_{\varvec{\xi }}(\varvec{t})v= \sum _{\varvec{a},\varvec{b}} \, {\mathcal {T}}(\varvec{t}^{ m})^{\varvec{a}}_{\varvec{b}} \;\left( \left( \overset{\rightarrow }{\prod \limits _{1< j\leqslant m} } \;\overset{\rightarrow }{\prod \limits _{1\leqslant i< j} }\;\ \overset{[j\; i]}{\mathbb {R}}(\varvec{t}^j,\varvec{t}^i)\right) \;\overset{[m-1]}{\mathbb {T}}(\varvec{t}^{m-1})\ldots \overset{[1]}{\mathbb {T}}(\varvec{t}^{ 1})\right) ^{\varvec{\ell }_1}_{\varvec{\ell }_2(\varvec{a})} \\&\times \left( \overset{[m+1]}{\mathbb {T}}(\varvec{t}^{m+1})\ldots \overset{[n-1]}{\mathbb {T}}(\varvec{t}^{n-1}) \left( \overset{\rightarrow }{\prod \limits _{m+1\leqslant j \leqslant n-1} }\;\;\overset{\rightarrow }{\prod \limits _{m\leqslant i<j} }\overset{[j\; i]}{\mathbb {R}}(\varvec{t}^j,\varvec{t}^i)\right) \right) ^{\varvec{\ell }_2(\varvec{b})}_{ \varvec{\ell }_3}v, \end{aligned}\end{aligned}$$

where the sequences $\varvec{a},\varvec{b}$ are as in (3.4) and

$$\begin{aligned}\begin{aligned} \varvec{\ell }_1&=(\varvec{1}^{\xi _1},\ldots ,\varvec{(m-1)}^{\xi _{m-1}},\varvec{m}^{\xi _m},(\varvec{m+1})^{\xi _m+1},\ldots ,(\varvec{n-1})^{\xi _n}),\\ \varvec{\ell }_2(\varvec{a})&=(\varvec{2}^{\xi _1},\ldots ,\varvec{m}^{\xi _{m-1}},\varvec{a},(\varvec{m+1})^{\xi _m+1},\ldots ,(\varvec{n-1})^{\xi _n}),\\ \varvec{\ell }_3&=(\varvec{2}^{\xi _1},\ldots ,\varvec{m}^{\xi _{m-1}},\varvec{(m+1)}^{\xi _m},(\varvec{m+2})^{\xi _m+1},\ldots ,\varvec{n}^{\xi _n}). \end{aligned} \end{aligned}$$

To prove formula (3.6), we take the definition of $\mathbb {B}_{\varvec{\xi }}(\varvec{t})v$ following from formulas (2.8), (2.9), and observe that using the Yang–Baxter equation, one can express $\mathbb {B}_{\varvec{\xi }}(\varvec{t})v$ as follows:

$$\begin{aligned} \mathbb {B}_{\varvec{\xi }}(\varvec{t})v=\left( \left( \overset{\rightarrow }{\prod \limits _{1\leqslant i< j\leqslant m} }\overset{[j\; i]}{\mathbb {R}}\right) \overset{[m]}{\mathbb {T}}\;\overset{[m-1]}{\mathbb {T}}\ldots \overset{[1]}{\mathbb {T}}\;\overset{[m+1]}{\mathbb {T}}\ldots \overset{[n-1]}{\mathbb {T}}\left( \overset{\rightarrow }{\prod \limits _{m+1\leqslant j \leqslant n-1} }\;\overset{\rightarrow }{\prod \limits _{1\leqslant i<j} }\overset{[j\; i]}{\mathbb {R}}\right) \right) ^{ \varvec{\ell }_1}_{ \varvec{\ell }_3}v. \end{aligned}$$

In detail, that gives

3.7 $$\begin{aligned} \mathbb {B}_{\varvec{\xi }}(\varvec{t})v= &   \sum _{\varvec{a},\varvec{b},\varvec{x},\varvec{y},\varvec{z}} \, {\mathcal {T}}(\varvec{t}^{ m})^{\varvec{a}}_{\varvec{b}} \;\left( \overset{\rightarrow }{\prod \limits _{1< j\leqslant m} } \;\overset{\rightarrow }{\prod \limits _{1\leqslant i< j} }\;\ \overset{[j\; i]}{\mathbb {R}} (\varvec{t}^j,\varvec{t}^i)\right) ^{\varvec{\ell }_1}_{\varvec{\ell }_4(\varvec{y},\varvec{a})} \; {\mathcal {T}}(\varvec{t}^{m-1})^{\varvec{y}^{(m-1)}}_{\varvec{x}^{(m-1)}} \ldots {\mathcal {T}}(\varvec{t}^1)^{\varvec{y}^{(1)}}_{\varvec{x}^{(1)}}\nonumber \\  &   \times {\mathcal {T}}(\varvec{t}^{m+1})^{\bullet }_{\varvec{z}^{(m+1)}} \ldots {\mathcal {T}}(\varvec{t}^{n-1})^{\bullet }_{\varvec{z}^{(n-1)}} \left( \overset{\rightarrow }{\prod \limits _{m+1\leqslant j \leqslant n-1} }\;\;\overset{\rightarrow }{\prod \limits _{1\leqslant i<j} }\overset{[j\; i]}{\mathbb {R}}(\varvec{t}^j,\varvec{t}^i)\right) ^{\varvec{\ell }_5(\varvec{x},\varvec{b},\varvec{z})}_{ \varvec{\ell }_3}v, \end{aligned}$$

where

$$\begin{aligned} \begin{aligned}&\varvec{x}=(\varvec{x}^{(1)},\ldots ,\varvec{x}^{(m-1)}), \qquad \varvec{y}=(\varvec{y}^{(1)},\ldots ,\varvec{y}^{(m-1)}) , \qquad \varvec{z}=(\varvec{z}^{(m+1)},\ldots , \varvec{z}^{(n-1)}),\\&\varvec{x}^{(l)}=(x^l_1,\ldots ,x^l_{\xi _l}),\qquad \varvec{y}^{(l)}=(y^l_1,\ldots ,y^l_{\xi _l}), \qquad \varvec{z}^{(l)}=(z^l_1,\ldots ,z^l_{\xi _l}), \\&{\mathcal {T}}(\varvec{t}^s)^{\varvec{y^{(s)}}}_{\varvec{x^{(s)}}}=T(t^s_1)^{y_1^s}_{x_1^s}\,T(t^s_2)^{y_2^s}_{x_2^s}\,\ldots \,T(t^s_{\xi _s})^{y_{\xi _s}^s}_{x_{\xi _s}^s},\qquad {\mathcal {T}}(\varvec{t}^s)^{\bullet }_{\varvec{z^{(s)}}}=T(t^s_1)^{s+1}_{z_1^s}\,T(t^s_2)^{s+1}_{z_2^s}\,\ldots \\&\qquad \,T(t^s_{\xi _s})^{s+1}_{z_{\xi _s}^s}, \end{aligned} \end{aligned}$$

$$\begin{aligned}\begin{aligned}&\varvec{\ell }_4(\varvec{y},\varvec{a})=\bigl (\varvec{y}^{(1)} ,\ldots ,\varvec{y}^{(m-1)} ,\varvec{a},(\varvec{m+1})^{\xi _m+1},\ldots ,(\varvec{n-1})^{\xi _n}\bigr ),\\&\varvec{\ell }_5(\varvec{x},\varvec{b},\varvec{z})=\bigl (\varvec{x}^{(1)} ,\ldots ,\varvec{x}^{(m-1)} ,\varvec{b}, \varvec{z}^{(m+1)},\ldots ,\varvec{z}^{(n-1)}\bigr )\;, \end{aligned}\end{aligned}$$

and the sum is taken over sequences $\varvec{a},\varvec{b},\varvec{x},\varvec{y},\varvec{z}$ with entries belonging to $\{1,\ldots , n\}$.

The next step is to show that for every $j=m+1,\ldots , n-1$, the product $\overset{\rightarrow }{\prod \nolimits _{1\leqslant i<j} }\overset{[j\; i]}{\mathbb {R}}$ in the second line of formula (3.7) can be truncated to $\overset{\rightarrow }{\prod \nolimits _{m\leqslant i<j} }\overset{[j\; i]}{\mathbb {R}}$ and the sum over $\varvec{x}=(\varvec{x}^{(1)},\ldots ,\varvec{x}^{(m-1)})$ reduces to a single term with $\varvec{x}=(\varvec{2}^{\xi _1},\ldots ,\varvec{m}^{\xi _{m-1}})$. This can be done by induction on *j*. The key idea is to combine two observations. First, since *v* is a weight singular vector, we have $z^s_k\geqslant s$ for all $s=m+1,\ldots , n-1$, and $k=1,\ldots ,\xi _s$. And second, the entries of *R*-matrix (2.1) have the property $R^{a\,b}_{c\,d}=\delta _{ac}\delta _{bd}$ if $b>c$, see formula (2.2). We have worked out this reasoning in detail for the $\mathfrak {gl}_4$-case in [6].

After the last step, formula (3.7) becomes

3.8 $$\begin{aligned} \begin{aligned} \mathbb {B}_{\varvec{\xi }}(\varvec{t})v&= \sum _{\varvec{a},\varvec{b}, \varvec{y},\varvec{z}} \, {\mathcal {T}}(\varvec{t}^{ m})^{\varvec{a}}_{\varvec{b}} \;\left( \overset{\rightarrow }{\prod \limits _{1< j\leqslant m} } \;\overset{\rightarrow }{\prod \limits _{1\leqslant i< j} }\;\ \overset{[j\; i]}{\mathbb {R}} (\varvec{t}^j,\varvec{t}^i)\right) ^{\varvec{\ell }_1}_{\varvec{\ell }_4(\varvec{y},\varvec{a})} \; {\mathcal {T}}(\varvec{t}^{m-1})^{\varvec{y}^{(m-1)}}_{\varvec{2}^{\xi _{m-1}}} \ldots {\mathcal {T}}(\varvec{t}^1)^{\varvec{y}^{(1)}}_{\varvec{2}^{ \xi _1}} \\&\times {\mathcal {T}}(\varvec{t}^{m+1})^{\bullet }_{\varvec{z}^{(m+1)}} \ldots {\mathcal {T}}(\varvec{t}^{n-1})^{\bullet }_{\varvec{z}^{(n-1)}} \left( \overset{\rightarrow }{\prod \limits _{m+1\leqslant j \leqslant n-1} }\;\;\overset{\rightarrow }{\prod \limits _{1\leqslant i<j} }\overset{[j\; i]}{\mathbb {R}}(\varvec{t}^j,\varvec{t}^i)\right) ^{\varvec{\ell }_6(\varvec{b},\varvec{z})}_{ \varvec{\ell }_3}v, \end{aligned}\end{aligned}$$

where

$$\begin{aligned} \varvec{\ell }_6(\varvec{b},\varvec{z}) =\bigl (\varvec{2}^{\xi _1} ,\ldots ,\varvec{m}^{\xi _{m-1}} ,\varvec{b}, \varvec{z}^{(m+1)},\ldots ,\varvec{z}^{(n-1)}\bigr )\;. \end{aligned}$$

Formula (2.2) for entries of the *R*-matrix implies that the entry $\bigl (\overset{\rightarrow }{\prod \nolimits _{1< j\leqslant m} } \;\overset{\rightarrow }{\prod \nolimits _{1\leqslant i< j} }\;\ \overset{[j\; i]}{\mathbb {R}} \;\bigr )^{\varvec{\ell }_1}_{\varvec{\ell }_4(\varvec{y},\varvec{a})}$ equals zero unless $a_i\in \{1,2,\ldots , m\}$ for all $i=1,\ldots ,\xi _m$, and the entry $\bigl (\overset{\rightarrow }{\prod \nolimits _{m+1\leqslant j \leqslant n-1} }\;\;\overset{\rightarrow }{\prod \nolimits _{1\leqslant i<j} }\overset{[j\; i]}{\mathbb {R}} \;\bigr )^{\varvec{\ell }_6(\varvec{b},\varvec{z})}_{ \varvec{\ell }_3}$ equals zero unless $b_i\in \{m+1,m+2,\ldots ,n\}$ for all $i=1,\ldots ,\xi _m$. Therefore, the sum over $\varvec{a},\varvec{b}$ in formula (3.8) reduces to the same range of summation variables as in formula (3.6). Furthermore, the sums over $\varvec{y}$ and $\varvec{z}$ in (3.8) can be evaluated,

$$\begin{aligned}\begin{aligned} \sum _{ \varvec{y} } \left( \overset{\rightarrow }{\prod \limits _{1< j\leqslant m} } \;\overset{\rightarrow }{\prod \limits _{1\leqslant i< j} }\;\ \overset{[j\; i]}{\mathbb {R}} (\varvec{t}^j,\varvec{t}^i)\right) ^{\varvec{\ell }_1}_{\varvec{\ell }_4(\varvec{y},\varvec{a})} \; {\mathcal {T}}(\varvec{t}^{m-1})^{\varvec{y}^{(m-1)}}_{\varvec{m}^{\xi _{m-1}}} \ldots {\mathcal {T}}(\varvec{t}^1)^{\varvec{y}^{(1)}}_{\varvec{2}^{ \xi _1}} \\ \quad =\left( \left( \overset{\rightarrow }{\prod \limits _{1< j\leqslant m} } \;\overset{\rightarrow }{\prod \limits _{1\leqslant i< j} }\;\ \overset{[j\; i]}{\mathbb {R}}(\varvec{t}^j,\varvec{t}^i)\right) \;\overset{[m-1]}{\mathbb {T}}(\varvec{t}^{m-1})\ldots \overset{[1]}{\mathbb {T}}(\varvec{t}^{ 1})\right) ^{\varvec{\ell }_1}_{\varvec{\ell }_2(\varvec{a})} \end{aligned}\end{aligned}$$

and

$$\begin{aligned} \begin{aligned} \sum _{ \varvec{z} } {\mathcal {T}}(\varvec{t}^{m+1})^{\bullet }_{\varvec{z}^{(m+1)}} \ldots {\mathcal {T}}(\varvec{t}^{n-1})^{\bullet }_{\varvec{z}^{(n-1)}} \left( \overset{\rightarrow }{\prod \limits _{m+1\leqslant j \leqslant n-1} }\;\;\overset{\rightarrow }{\prod \limits _{1\leqslant i<j} }\overset{[j\; i]}{\mathbb {R}}(\varvec{t}^j,\varvec{t}^i)\right) ^{\varvec{\ell }_6( \varvec{b},\varvec{z})}_{ \varvec{\ell }_3} \\ \quad = \left( \overset{[m+1]}{\mathbb {T}}(\varvec{t}^{m+1})\ldots \overset{[n-1]}{\mathbb {T}}(\varvec{t}^{n-1}) \left( \overset{\rightarrow }{\prod \limits _{m+1\leqslant j \leqslant n-1} }\;\;\overset{\rightarrow }{\prod \limits _{m\leqslant i<j} }\overset{[j\; i]}{\mathbb {R}}(\varvec{t}^j,\varvec{t}^i)\right) \right) ^{\varvec{\ell }_2(\varvec{b})}_{ \varvec{\ell }_3}. \end{aligned} \end{aligned}$$

Thus, we transformed formula (3.8) to formula (3.6). Proposition 3.1 is proved. $\square$

### Remark

The statement of Proposition 3.1 holds for any singular vector *v* in any $Y(\mathfrak {g l}_{n})$-module.

### Remark

For $\xi _m=0$, Proposition 3.1 takes the form

3.9 $$\begin{aligned} \mathbb {B}_{\varvec{\xi }}(\varvec{t})v = \phi _m \left( \mathbb {B}_{\varvec{\dot{\xi }} }^{\langle m\rangle }(\dot{\varvec{t}})\right) \; \psi _m\left( \mathbb {B}_ {\varvec{\ddot{\xi }}}^{\langle n-m\rangle }(\ddot{\varvec{t}})\right) v. \end{aligned}$$

Notice that the transformation of formula (3.8) to formula (3.6) in the case $\xi _m=0$ remains nontrivial.

## Weight functions

Fix a positive integer *M*. Consider a collection of nonnegative integers $\varvec{\lambda }=(\lambda _1,\ldots ,\lambda _m)$ such that $\sum _{k=1}^{m} \lambda _k=M$. Set $\lambda ^{s}=\sum _{k=1}^s \lambda _k, \quad s=1, \ldots , m$. Introduce the variables $u_1^{s},\ldots , u_{\lambda ^{s}}^{s}$, $s=1, \ldots , m$. Denote $\varvec{u}^{s}=\left( u_1^{s}, \ldots , u_{\lambda ^{s}}^{s}\right)$ and $\varvec{ {u}}=\left( \varvec{u}^{1}, \ldots , \varvec{u}^{m-1}\right)$. Let $\varvec{J} = (J_1,\ldots , J_m)$ be a partition of $\{1,\ldots ,M\}$ into disjoint subsets $J_1,\ldots , J_m$ such that $\left| J_k\right| =\lambda _k$, $k=1,\ldots ,m$.

Similarly, consider a collection of nonnegative integers $\varvec{\mu }=(\mu _{m+1},\ldots ,\mu _{n})$, such that $\sum _{l=m+1}^n \mu _l~=M$. Set $\mu ^{r}=\sum _{k=r+1}^n \mu _k, \quad r=m, \ldots , n-1$. Introduce the variables $v_1^{r},\ldots , v_{\mu ^{r}}^{r}$, $r=m, \ldots , n-1$. Denote $\varvec{v}^{r}=\left( v_1^{r}, \ldots , v_{\mu ^{r}}^{r}\right)$ and $\varvec{v}=\left( \varvec{v}^{m+1}, \ldots , \varvec{v}^{n-1}\right)$. Let $\varvec{I}=\left( I_{m+1},\ldots , I_{n}\right)$ be a partition of $\{1, \ldots , M\}$ into disjoint subsets $I_{m+1},\ldots , I_{n}$ such that $\left| I_j\right| =\mu _j$, $j=m+1, \ldots , n$.

Given partitions $\varvec{I},\varvec{J}$ as above, we define rational functions $U_{\varvec{I}}(\varvec{v}; \varvec{v}^m )$ and $\widetilde{U}_{\varvec{J}}(\varvec{u}; \varvec{u}^m)$ that will be extensively used later in this paper. Set

4.1 $$\begin{aligned}  &   U_{\varvec{I}}(\varvec{v} ; \varvec{v}^m) \nonumber \\  &   \quad =\prod _{l=m+1}^{n-1} \prod _{a=1}^{\mu ^{l}}\left( \prod _{\begin{array}{c} c=1 \\ i_c^{(l- 1)}=i_a^{(l)} \end{array}}^{\mu ^{l-1}}\left( \dfrac{1}{v_a^{l}-v_c^{l-1}}\right) \prod _{\begin{array}{c} d=1 \\ i_d^{(l-1)}>i_a^{(l)} \end{array}}^{\mu ^{l-1}}\left( \dfrac{v_a^{l}-v_d^{l-1}+1}{v_a^{l}-v_d^{l-1}}\right) \prod _{b=a+1}^{\mu ^{l}} \frac{v_b^{l}-v_a^{l}+1}{v_b^{l}-v_a^{l}}\right) , \end{aligned}$$

where the numbers $i_c^{(l)}$ are defined as follows

$$\begin{aligned} \bigcup _{k=l+1}^n I_k=\{i_1^{(l)}<\ldots <i_{\mu ^{l}}^{(l)}\}. \end{aligned}$$

Similarly, set

4.2 $$\begin{aligned}  &   \widetilde{U}_{\varvec{J}}(\varvec{u} ; \varvec{u}^m)\nonumber \\  &   \quad =\prod _{l=2}^{m} \prod _{a=1}^{\lambda ^{l}}\left( \prod _{\begin{array}{c} c=1 \\ j_c^{(l-1)}<j_a^{(l)} \end{array}}^{\lambda ^{l-1}}\left( \dfrac{u_a^{l}-u_c^{l-1}+1}{u_a^{l}-u_c^{l-1}}\right) \prod _{\begin{array}{c} d=1 \\ j_d^{(l-1)}=j_a^{(l)} \end{array}}^{\lambda ^{l-1}}\left( \dfrac{1}{u_a^{l}-u_d^{l-1}}\right) \prod _{b=a+1}^{\lambda ^{l}} \frac{u_a^{l}-u_b^{l}+1}{u_a^{l}-u_b^{l}}\right) , \end{aligned}$$

where the numbers $j_c^{(l)}$ are defined as follows

$$\begin{aligned} \bigcup _{k=1}^l J_k=\{j_1^{(l)}<\ldots < j_{\lambda ^{l}}^{(l)}\}. \end{aligned}$$

For a function $f\left( x_1, \ldots , x_k\right)$ of some variables, denote

$$\begin{aligned} \operatorname {Sym}_{x_1, \ldots , x_k} f\left( x_1, \ldots , x_k\right) =\sum _{\sigma \in S_k} f\left( x_{\sigma (1)}, \ldots , x_{\sigma (k)}\right) . \end{aligned}$$

Introduce the weight functions $W_{\varvec{I}}(\varvec{v}; \varvec{v}^m )$ and $\widetilde{W}_{\varvec{J}}(\varvec{u};\varvec{u}^m )$

4.3 $$\begin{aligned} W_{\varvec{I}}(\varvec{v} ; \varvec{v}^m )=\operatorname {Sym}_{v_1^{m+1}, \ldots , v_{\mu ^{m+1}}^{m+1}} \ldots \operatorname {Sym}_{v_1^{n-1}, \ldots , v_{\mu ^{n-1}}^{n-1}} U_{\varvec{I}}(\varvec{v} ; \varvec{v}^m ), \end{aligned}$$

4.4 $$\begin{aligned} \widetilde{W}_{\varvec{J}}(\varvec{u} ; \varvec{u}^m )=\operatorname {Sym}_{u_1^{1}, \ldots , u_{\lambda ^{1}}^{1}} \ldots \operatorname {Sym}_{u_1^{m-1}, \ldots , u_{\lambda ^{m-1}}^{m-1}} U_{\varvec{J}}(\varvec{u} ; \varvec{u}^m ), \end{aligned}$$

For $\sigma \in S_M$ and $\varvec{J}=\left( J_1, \ldots , J_m\right)$, set $\sigma (\varvec{J})=\left( \sigma \left( J_1\right) , \ldots , \sigma \left( J_m\right) \right)$. Similarly, for $\varvec{I}=(I_m,\ldots ,I_n)$, $\sigma (\varvec{I})=\left( \sigma \left( I_m\right) , \ldots , \sigma \left( I_n\right) \right)$. For $a, b=1, \ldots , n$, let $s_{a, b}$ be the transposition of *a*, *b*.

Here is the main property of the weight functions, see for instance [15].

### Lemma 4.1

Let $$\varvec{z}=(z_1,\ldots ,z_{\xi _m})$$. Then,

$$\begin{aligned} W_{\varvec{I}}\left( \varvec{v}; z_1, \ldots , z_{a+1}, z_a, \ldots , z_{\xi _m} \right)= &   \frac{z_{a+1}-z_{a}}{z_{a+1}-z_{a}-1}\,W_{s_{a, a+1}(\varvec{I})}(\varvec{v};\varvec{z}) \,\\  &   -\,\frac{1}{z_{a+1}-z_{a}-1}\,W_{\varvec{I}}(\varvec{v}; \varvec{z}), \end{aligned}$$

$$\begin{aligned} \widetilde{W}_{\varvec{J}}\left( \varvec{u};z_1,\ldots ,z_{a+1},z_a, \ldots , z_{\xi _m}\right) \,= &   \frac{z_{a}-z_{a+1}}{z_{a}-z_{a+1}-1}\,\widetilde{W}_{s_{a, a+1}(\varvec{J})}(\varvec{u}; \varvec{z}) \,\\  &   -\,\frac{1}{z_{a}-z_{a+1}-1}\,\widetilde{W}_{\varvec{J}}(\varvec{u};\varvec{z}). \end{aligned}$$

This property provides us with the tool for the proof of the main result of this paper, Theorem 5.5.

## Main theorem

The main result of this paper is Theorem 5.5 formulated at the end of this section. It will be approached in several steps. We use the notation given in (3.3).

### Definition 1

For a collection $\varvec{a}=(a_1,\ldots , a_{\xi _m})$ such that $a_i\in \{1,2,\ldots , m\}$ for all $i=1,\ldots ,\xi _m$, we define a partition $\varvec{J}(\varvec{a})=(J_1,\ldots , J_m)$ of $\{1,\ldots ,\xi _m\}$ by the rule $J_l=\{j| a_j=l\}$. Denote $|\cup _{r=1}^s J_r|=\zeta _s$, so that we have $\xi _m=\zeta _m\geqslant \zeta _{m-1}\geqslant \ldots \geqslant \zeta _1\geqslant 0$. Denote $\varvec{\zeta }~=~(\zeta _{ 1},\ldots , \zeta _{m-1})$.

For a collection $\varvec{b}=\left( b_1, \ldots , b_{\xi _m}\right)$ such that $b_i\in \{m+1,m+2,\ldots ,n\}$ for all $i=1,\ldots ,\xi _m$, we define a partition $\varvec{I}(\varvec{b})=(I_{m+1},\ldots , I_{n})$ of $\{1,\ldots ,\xi _m\}$ by the rule $I_l=\{i| b_i=l\}$. Denote $|\cup _{r=s+1}^n I_r|=\eta _{s}$, so that we have $\xi _m=\eta _{m}\geqslant \eta _{m+1}\geqslant \ldots \geqslant \eta _{n-1}\geqslant 0$. Denote $\varvec{\eta }=(\eta _{m+1},\ldots ,\eta _{n-1})$.

### Lemma 5.1

The correspondences $\varvec{a}\mapsto \varvec{J}(\varvec{a})$ and $\varvec{b}\mapsto \varvec{I}(\varvec{b})$ are bijections.

### Proof

By inspection. $$\square $$

Consider $\varvec{\eta }\in \mathbb {Z}_{\geqslant 0}^{n-m-1}$, $\varvec{\zeta }\in \mathbb {Z}_{\geqslant 0}^{m-1}$, such that $\eta _i\leqslant \xi _i$ and $\zeta _j\leqslant \xi _j$ for all relevant *i*, *j*. Set

$$\begin{aligned} \begin{aligned} \varvec{t}^m&=(t_1^m, \ldots , t_{\xi _m}^m)\\ \ddot{\varvec{t}}_{[ \varvec{{\eta }}]}&=\left( t_1^{m+1}, \ldots , t_{\eta _{m+1}}^{m+1} ; \ldots ; t_1^{n-1}, \ldots , t_{\eta _{n-1}}^{n-1}\right) , \\ \ddot{\varvec{t}}_{(\varvec{\eta }, \varvec{\ddot{\xi }}]}&=\left( t_{\eta _{m+1}+1}^{m+1}, \ldots , t_{\xi _{m+1}}^{m+1} ; \ldots ; t_{\eta _{n-1}+1}^{n-1}, \ldots , t_{\xi _{n-1}}^{n-1}\right) ,\\ \dot{\varvec{t}}_{[\varvec{\dot{\xi }}-\varvec{\zeta }]}&=\left( t_1^1, \ldots , t_{\xi _1-\zeta _1}^1 ; \ldots ; t_1^{m-1}, \ldots , t_{\xi _{m-1}-\zeta _{m-1}}^{m-1}\right) , \\ \dot{\varvec{t}}_{(\varvec{\dot{\xi }}-\varvec{\zeta }, \varvec{\dot{\xi }}]}&=\left( t_{\xi _1-\zeta _1+1}^1, \ldots , t_{\xi _1}^1 ; \ldots ; t_{\xi _{m-1}-\zeta _{m-1}+1}^{m-1}, \ldots , t_{\xi _{m-1}}^{m-1}\right) . \end{aligned} \end{aligned}$$

### Lemma 5.2

For a given sequence $\varvec{b}=(b_1,\ldots ,b_{\xi _m})$, where $b_i\in \{m+1,m+2,\ldots ,n\}$, consider the corresponding partition $\varvec{I}(\varvec{b})$ and the decreasing sequence $\eta _m=\xi _m \geqslant \eta _{m+1}\geqslant \cdots \geqslant \eta _{n-1}$ as described in Definition 1. Denote $\varvec{\eta }=\left( \eta _{m+1}, \ldots , \eta _{n-1}\right) \in \mathbb {Z}_{\geqslant \ 0}^{n-m-1}$. Let $\tilde{v}$ be a vector, such that $T^a_c(u)\tilde{v}=0$, for $m\leqslant c<a\leqslant n$, and $T_{c}^c(u)\tilde{v}=(1+\Lambda ^{c}(u-x)^{-1})\tilde{v}$, $c=m,\ldots ,n$. Then for $\varvec{\ddot{\xi }}-\varvec{\eta }\in \mathbb {Z}^{n-m-1}_{\geqslant 0}$, we have

$$\begin{aligned}  &   \Bigl (\psi _{n-m}(\varvec{t}^{m})\left( \mathbb {B}_{\varvec{\ddot{\xi }}}^{\langle n-m\rangle }(\ddot{\varvec{t}})\right) \Bigr )^{\varvec{b}} \tilde{v} =\prod _{b=m+1}^{n-1} \frac{1}{\left( \xi _b-\eta _b\right) !}\;{\operatorname {Sym}}_{\ddot{\varvec{t}}}\left[ U_{\varvec{I}(\varvec{b})}\left( \varvec{\ddot{t}}_{[\varvec{\eta }]}, \varvec{t}^m\right) L_{\varvec{\eta },\varvec{\ddot{\xi }}}(\varvec{\ddot{t}},\varvec{t}^{ m}) \right] \tilde{v}, \end{aligned}$$

where $U_{\varvec{I}(\varvec{b})}\left( \varvec{\ddot{t}}_{[\varvec{\eta }]}, \varvec{t}^m \right)$ is given by formula (4.1) and

5.1 $$\begin{aligned} L_{\varvec{\eta },\varvec{\ddot{\xi }}}(\varvec{\ddot{t}}\,)\,  &   = \prod _{a=m+1}^{n-2} \prod _{i=1}^{\eta _{a+1}} \prod _{j=\eta _a+1}^{\xi _a} \frac{t_i^{a+1}-t_j^a+1}{t_i^{a+1}-t_j^a} \prod _{l=m+1}^{n-1} \prod _{i=1}^{\eta _l} \frac{t_i^l-x+\Lambda ^l}{t_i^l-x} \;\psi _{n-m}\nonumber \\    &   \left( \mathbb {B}_{\varvec{\ddot{\xi }}-\varvec{\eta } }^{\langle n-m\rangle } \left( \varvec{\ddot{t}}_{(\varvec{\eta }, \varvec{\ddot{\xi }}]}\right) \right) . \end{aligned}$$

If $$\,\varvec{\ddot{\xi }}-\varvec{\eta }\not \in \mathbb {Z}^{n-m-1}_{\geqslant 0}$$, then $$\Bigl (\psi _{n-m}(\varvec{t}^{m})\left( \mathbb {B}_ {\ddot{\varvec{\xi }}}^{\langle n-m\rangle }(\ddot{\varvec{t}})\right) \Bigr )^{\varvec{b}} \tilde{v} =0$$.

### Proof

The statement coincides with Lemma 4.3 in [23] up to a change of notation. $\square$

### Lemma 5.3

For a given sequence ${\varvec{a}}=(a_1,\ldots ,a_{\xi _m})$, where $a_i\in \{1,2,\ldots , m\}$, consider the corresponding partition $\varvec{J}(\varvec{a})$ and the increasing sequence $\zeta _1 \leqslant \zeta _2\leqslant \cdots \leqslant \zeta _{m-1}\leqslant \zeta _{m}=\xi _m$ as described Definition 1. Denote $\varvec{\zeta }=\left( \zeta _1, \ldots , \zeta _{m-1}\right) \in \mathbb {Z}_{\geqslant 0}^{m-1}$. Let $\hat{v}$ be a vector, such that $T^c_b(u)\hat{v}=0$, for $1\leqslant b<c\leqslant m$, and $T_{b}^b(u)\hat{v}=(1+\Lambda ^{b}(u-x)^{-1})\hat{v}$, $b= 1,\ldots ,m$. Then, for $\varvec{\dot{\xi }-\varvec{\zeta }}\in \mathbb {Z}^{m-1}_{\geqslant 0}$, we have

$$\begin{aligned} \Bigl (\phi _m(\varvec{t}^m)\left( \mathbb {B}_{\varvec{\dot{\xi } }}^{\langle m\rangle }(\dot{\varvec{t}})\right) \Bigr )^{\varvec{a}} \hat{v} \,=\,\prod _{l=1}^{m} \frac{1}{\left( \xi _l-\zeta _l\right) !}\; {\operatorname {Sym}}_{\varvec{\dot{t}}} \left[ \widetilde{U}_{\varvec{J}(\varvec{a})}(\varvec{\dot{t}}_{(\varvec{\dot{\xi }}-\varvec{\zeta },\varvec{\dot{\xi }}]},\varvec{t}^m) \widetilde{L}_{\varvec{\zeta },\varvec{\dot{\xi }}} (\varvec{\dot{t}}\,)\right] \hat{v}, \end{aligned}$$

where $\widetilde{U}_{\varvec{J}(\varvec{a})}(\varvec{\dot{t}}_{(\varvec{\dot{\xi }}-\varvec{\zeta },\varvec{\dot{\xi }}]},{\varvec{t}}^m )$ is given by formula (4.2) and

5.2 $$\begin{aligned} \begin{aligned} \widetilde{L}_{\varvec{\zeta },\varvec{\dot{\xi }}} (\varvec{\dot{t}}\,)\,& = \prod _{a=1}^{m-2}\,\prod _{i=1}^{\xi _{a+1}-\eta _{a+1}}\!\!\prod _{j=\xi _a-\eta _a+1}^{\xi _a} \frac{t_i^{a+1}-t_j^a+1}{t_i^{a+1}-t_j^a}\\& \times \prod _{l=1}^{m-1}\,\prod _{i=0}^{\eta ^l-1}\,\frac{t_{\xi _l-i}^l-x+\Lambda ^{l+1}}{t_{\xi _l-i}^l-x} \;\phi _m\left( \mathbb {B}_{\varvec{\dot{\xi }}-\varvec{\zeta }}^{\langle m\rangle }\left( \varvec{\dot{t}}_{[\varvec{\dot{\xi }}-\varvec{\zeta }]}\right) \right) . \end{aligned}\end{aligned}$$

If $$\varvec{\dot{\xi }-\varvec{\zeta }}\not \in \mathbb {Z}^{m-1}_{\geqslant 0}$$, then $$\Bigl (\phi _m(\varvec{t}^m)\left( \mathbb {B}_{\varvec{\dot{\xi } }}^{\langle m\rangle }(\dot{\varvec{t}})\right) \Bigr )^{\varvec{a}} \hat{v}=0$$.

### Proof

The proof is similar to the proof of Lemma 4.3 in [23] with Corollary 3.4 there replaced by Corollary 3.2 *op.cit.* $\square$

Consider collections $\varvec{q}=(q_{sp}$: $s=m+1,\ldots , n,$ $p=1,\ldots , m$) of nonnegative integers and introduce

5.3 $$\begin{aligned} \begin{aligned}&\eta _k(\varvec{q})= \sum _{s=k+1}^n\sum _{p=1}^m q_{sp}, \quad k=m+1,\ldots , n-1,\\&\zeta _l(\varvec{q})= \sum _{s=m+1}^n\sum _{p=1}^l q_{sp}, \quad l=1,\ldots ,m-1. \end{aligned}\end{aligned}$$

Given $\varvec{\xi }=(\xi _1,\ldots ,\xi _{n-1})$, define a set

5.4 $$\begin{aligned} \mathcal {Q}_{m,n}=\Bigl \{\varvec{q}=(q_{sp}): \;\sum _{s=m+1}^n\sum _{p=1}^m q_{sp}=\xi _m,\quad \eta _k(\varvec{q})\leqslant \xi _k, \quad \zeta _l(\varvec{q})\leqslant \xi _l, \; \,{\text { for all }}\, k,l \Bigr \}. \end{aligned}$$

In addition, for any $\varvec{q}\in \mathcal {Q}_{m,n}$, we consider the set $\mathcal {S}_{\varvec{q}}$ of all pairs $(\varvec{I},\varvec{J})$ of partitions $\varvec{I}=(I_{m+1},\ldots , I_{n})$, $\,\varvec{J}=(J_1,\ldots , J_m)$ of $\{1,\ldots ,\xi _m\}$ with given cardinalities of intersections,

5.5 $$\begin{aligned} \mathcal {S}_{\varvec{q}}=\bigl \{\;(\varvec{I},\varvec{J}):\;\; \left| I_s\cap J_p\right| =q_{sp}, \;\;s=m+1,\ldots ,n,\;p=1,\ldots ,m\;\bigr \}. \end{aligned}$$

Notice that,

$$\begin{aligned} \left| \cup _{r=k+1}^n I_r\right| =\eta _{k}(\varvec{q}),\quad \left| \cup _{r=1}^l J_r\right| =\zeta _l (\varvec{q}). \end{aligned}$$

### Proposition 5.4

Let $v\in V(x)$ be a weight singular vector of $\mathfrak {gl}_n$-weight $(\Lambda _1,\ldots \Lambda _n)$. Then,

5.6 $$\begin{aligned} \begin{aligned} \mathbb {B}_{\varvec{\xi }}(\varvec{t})v\,= \,&\prod _{l=1}^{\xi _m}\dfrac{1}{t_{l}^m-x}\, \sum _{\varvec{q}\in \mathcal {Q}_{m,n}}\,\prod _{i=m+1}^n\,\prod _{j=1}^m\,e^{q_{ij}}_{ij}\, \prod _{a=1}^{m-1}\frac{1}{\left( \xi _a-\zeta _a\right) !}\; \prod _{b=m+1}^{n-1} \frac{1}{\left( \xi _b-\eta _b\right) !} \\& \times  {\operatorname {Sym}}_{\varvec{\ddot{t}}} \; { {\operatorname {Sym}_{\varvec{\dot{t}}}}} \Biggl [L_{\varvec{\eta },\varvec{\ddot{\xi }}}(\varvec{\ddot{t}}\,)\,\widetilde{L}_{\varvec{\zeta },\varvec{\dot{\xi }}} (\varvec{\dot{t}}\,)\!\sum _{(\varvec{I},\varvec{J}) \in \mathcal {S}_{\varvec{q}}}\!\widetilde{U}_{\varvec{J}}(\varvec{\dot{t}}_{(\varvec{\dot{\xi }}-\varvec{\zeta },\varvec{\dot{\xi }}]},\varvec{t}^m ) U_{\varvec{I}}\left( \varvec{\ddot{t}}_{[\varvec{\eta }]}, \varvec{t}^m \right) \Biggr ]\,v,\!\! \end{aligned}\end{aligned}$$

where the sequences $\varvec{\eta }=\left( \eta _{m+1}, \ldots , \eta _{n-1}\right)$ and $\varvec{\zeta }=\left( \zeta _1, \ldots , \zeta _{m-1}\right)$, are given by the rule

$$\begin{aligned}\eta _k=\eta _k(\varvec{q}),\quad \zeta _l= \zeta _l(\varvec{q}), \quad k=m+1,\ldots , n-1, \quad l=1,\ldots ,m-1.\end{aligned}$$

### Proof

Taking formula (3.4) for $\mathbb {B}_{\varvec{\xi }}(\varvec{t})v$, we apply Lemma 5.2 to the expression $\Bigl (\psi _m(\varvec{t}^{m})\left( \mathbb {B}_{\varvec{\ddot{\xi }}}^{\langle n-m\rangle }(\ddot{\varvec{t}})\right) \Bigr )^{\varvec{b}}v$. Next we observe that the vector $\psi _{n-m}\left( \mathbb {B}_{\varvec{\ddot{\xi }}-\varvec{\eta } }^{\langle n-m \rangle } \left( \varvec{\ddot{t}}_{(\varvec{\eta }, \varvec{\ddot{\xi }}]}\right) \right) v$ in the right-hand side of (5.1) satisfies the conditions for the vector $\hat{v}$ in Lemma 5.3. Applying Lemma 5.3 to the expression $\Bigl (\phi _m(\varvec{t}^m)\left( \mathbb {B}_{\varvec{\dot{\xi } }}^{\langle m\rangle }(\dot{\varvec{t}})\right) \Bigr )^{\varvec{a}}\,\psi _{n\!-\!m}\!\left( \mathbb {B}_{\varvec{\ddot{\xi }}\!-\!\varvec{\eta } }^{\langle n\!-\!m \rangle } \bigl (\varvec{\ddot{t}}_{(\varvec{\eta }, \varvec{\ddot{\xi }}]}\bigr )\right) v$, we obtain

$$\begin{aligned}  &   \mathbb {B}_{\varvec{\xi }}(\varvec{t})v = \sum _{\varvec{a},\varvec{b}} \Bigl ( {\mathcal {T}} (\varvec{t}^{m})\Bigr )^{\varvec{a}}_{\varvec{b}} \; {\operatorname {Sym}}_{\varvec{\ddot{t}}} \;\\  &   { {\operatorname {Sym}}_{\varvec{\dot{t}}}} \Biggl [ \widetilde{U}_{\varvec{J}(\varvec{a})}(\varvec{\dot{t}}_{(\varvec{\dot{\xi }}-\varvec{\zeta },\varvec{\dot{\xi }}]},\varvec{t}^m ) U_{\varvec{I}(\varvec{b})}\left( \varvec{\ddot{t}}_{[\varvec{\eta }]}, \varvec{t}^m \right) L_{\varvec{\eta },\varvec{\ddot{\xi }}}(\varvec{\ddot{t}}\,) \widetilde{L}_{\varvec{\zeta },\varvec{\dot{\xi }}} (\varvec{\dot{t}}\,)\Biggr ]v, \end{aligned}$$

where $\Bigl ( {\mathcal {T}} (\varvec{t}^{m})\Bigr )^{\varvec{a}}_{\varvec{b}}$ is given by (3.5), while the partitions $\varvec{J}(\varvec{a}), \varvec{I}(\varvec{b})$ and the sequences $\varvec{\eta }=(\eta _{m+1}, \ldots ,\eta _{n-1})$, $\varvec{\zeta }=(\zeta _{1},\ldots ,\zeta _{m-1})$ are described in Definition 1.

On the next step, we use the bijection between the sequences $\varvec{a},\varvec{b}$ and the partitions $\varvec{I},\varvec{J}$, see Lemma 5.1, to rewrite the sum $\sum _{\varvec{a},\varvec{b}}$ as the sum $\sum _{\varvec{I},\varvec{J}}$ over pairs of partitions. The latter sum can be further written as the double sum $\sum _{\varvec{q}\in \mathcal {Q}_{m,n}}\sum _{ (\varvec{I},\varvec{J})\in \mathcal {S}_{\varvec{q}}}$ over collections $\varvec{q}\in \mathcal {Q}_{m,n}$ and pairs of partitions $(\varvec{I},\varvec{J})\in \mathcal {S}_{\varvec{q}}$. Thus, we obtain

$$\begin{aligned}\begin{aligned} \mathbb {B}_{\varvec{\xi }}(\varvec{t})v\,& =\! \sum _{\varvec{q}\in \mathcal {Q}_{m,n}}\;\sum _{(\varvec{I},\varvec{J}) \in \mathcal {S}_{\varvec{q}}}\Bigl ( {\mathcal {T}} (\varvec{t}^{m})\Bigr )^{\varvec{a}(\varvec{J})}_{\varvec{b}(\varvec{I})}\times  \\& \times \,{\operatorname {Sym}}_{\varvec{\ddot{t}}} \; { {\operatorname {Sym}}_{\varvec{\dot{t}}}} \Biggl [ \widetilde{U}_{\varvec{J}(\varvec{a})}(\varvec{\dot{t}}_{(\varvec{\dot{\xi }}-\varvec{\zeta },\varvec{\dot{\xi }}]},\varvec{t}^m ) U_{\varvec{I}(\varvec{b})}\left( \varvec{\ddot{t}}_{[\varvec{\eta }]}, \varvec{t}^m \right) L_{\varvec{\eta },\varvec{\ddot{\xi }}}(\varvec{\ddot{t}}\,) \widetilde{L}_{\varvec{\zeta },\varvec{\dot{\xi }}} (\varvec{\dot{t}}\,)\Biggr ]v\,, \end{aligned}\end{aligned}$$

where $\varvec{a}(\varvec{J})$, $\varvec{b}(\varvec{I})$ are the sequences corresponding to the partitions $\varvec{I}$, $\varvec{J}$.

Finally, we observe that in the module *V*(*x*) we have $\Bigl ( {\mathcal {T}} (\varvec{t}^{m})\Bigr )^{\varvec{a}}_{\varvec{b}} =\prod _{i,j} e^{q_{ij}}_{ji} \prod _{l=1}^{\xi _m}\dfrac{1}{t_{l}^m-x}$. The last expression and the product $L_{\varvec{\eta },\varvec{\ddot{\xi }}}(\varvec{\ddot{t}}\,) \widetilde{L}_{\varvec{\zeta },\varvec{\dot{\xi }}} (\varvec{\dot{t}}\,)$ depend only on the collection $\varvec{q}$ and can be moved out of the sum $\sum _{ (\varvec{I},\varvec{J}) \in \mathcal {S}_{\varvec{q}}}$. Proposition 5.4 is proved. $\square$

The next theorem is the main result of this paper. It will be proved in Sect. 6.

### Theorem 5.5

Let $v\in V(x)$ be a weight singular vector of $\mathfrak {gl}_n$-weight $(\Lambda _1,\ldots \Lambda _n)$ and $\varvec{\xi }=(\xi _1,\ldots ,\xi _{n-1})$ be a collection of nonnegative integers. Then,

5.7 $$\begin{aligned} \begin{aligned} \!\mathbb {B}_{\varvec{\xi }}(\varvec{t})v\,= \,&\prod _{l=1}^{\xi _m}\,\dfrac{1}{t_{l}^m-x} \sum _{\varvec{q}\in \mathcal {Q}_{m,n}}\,\prod _{i=m+1}^n\,\prod _{j=1}^m\,\dfrac{e^{q_{ij}}_{ij}}{q_{ij}!}\;\, \prod _{a=1}^{m-1} \frac{1}{\left( \xi _a-\zeta _a\right) !}\; \prod _{b=m+1}^{n-1} \frac{1}{\left( \xi _b-\eta _b\right) !} \\& \times {{\operatorname {Sym}}_{{\varvec{t}}^m}} \,{{\operatorname {Sym}}_{\dot{\varvec{t}}}}\,{\operatorname {Sym}}_{\ddot{\varvec{t}}} \left[ L_{\varvec{\eta },\varvec{\ddot{\xi }}}(\varvec{\ddot{t}}\,) \widetilde{L}_{\varvec{\zeta },\varvec{\dot{\xi }}} (\varvec{\dot{t}}\,) \; \widetilde{U}_{\varvec{J}_0}(\varvec{\dot{t}}_{(\varvec{\dot{\xi }}-\varvec{\zeta },\varvec{\dot{\xi }}]},\check{\varvec{t}}^m ) U_{\varvec{\check{I}}_0}\left( \varvec{\ddot{t}}_{[\varvec{\eta }]}, \varvec{t}^m \right) \Phi (\varvec{t}^m)\right] v. \end{aligned}\end{aligned}$$

Here the set $\mathcal {Q}_{m,n}$ is given by (5.4), $\varvec{I_0}=(I_{m+1},\ldots , I_{n})$, $\varvec{J_0}=(J_1,\ldots , J_m)$ is any pair of partitions of $\{1,\ldots ,\xi _m\}$ such that $(\varvec{I}_0,\varvec{J}_0)\in \mathcal {S}_{\varvec{q}}$, see (5.5), $\varvec{\check{I}}_0=(\sigma _0(I_{m+1}),\ldots ,\sigma _0 (I_{n}))$, where $\sigma _0\in S_{\xi _m}$ is the longest permutation, $\sigma _0(i)=\xi _m-i+1$, the sequences $\varvec{\eta }=\left( \eta _{m+1}, \ldots , \eta _{n-1}\right)$, $\varvec{\zeta }=\left( \zeta _1, \ldots , \zeta _{m-1}\right)$, are given by the rule $\eta _k=\eta _k(\varvec{q}),\quad \zeta _l= \zeta _l(\varvec{q})$ for all *k*, *l*, see (5.3), the functions $L_{\varvec{\eta },\varvec{\ddot{\xi }}}(\varvec{\ddot{t}},\varvec{t}^m )$, $\widetilde{L}_{\varvec{\zeta },\varvec{\dot{\xi }}} (\varvec{\dot{t}},\varvec{t}^m )$ are given by (5.1),(5.2), the functions $U_{\varvec{\check{I}}_0(\varvec{b})}\left( \varvec{\ddot{t}}_{[\varvec{\eta }]}, \varvec{t}^m \right)$, $\widetilde{U}_{\varvec{J}_0(\varvec{a})}(\varvec{\dot{t}}_{(\varvec{\dot{\xi }}-\varvec{\zeta },\varvec{\dot{\xi }}]},\check{\varvec{t}}^m )$ are given by (4.1), (4.2), $\check{\varvec{t}}^m=(t_{\xi _m}^m,\ldots , t_{1}^m)$ and

$$\begin{aligned} \Phi (\varvec{t}^m)= \prod _{1\leqslant a<b\leqslant \xi _m} \dfrac{t^m_a-t^m_b-1}{t^m_a-t^m_b} . \end{aligned}$$

### Remark

For $\xi _m=0$, we have $\varvec{\eta }=(0,\ldots ,0)$, $\varvec{\zeta }=(0,\ldots ,0)$. Then   $\widetilde{U}_{\varvec{J}_0(\varvec{a})}(\varvec{\dot{t}}_{(\varvec{\dot{\xi }}-\varvec{\zeta },\varvec{\dot{\xi }}]},\check{\varvec{t}}^m )=1$, $U_{\varvec{\check{I}}_0(\varvec{b})}\left( \varvec{\ddot{t}}_{[\varvec{\eta }]}, \varvec{t}^m \right) =1$, $L_{\varvec{\eta },\varvec{\ddot{\xi }}}(\varvec{\ddot{t}}\,)=\psi _{n-m}\left( \mathbb {B}_{\varvec{\ddot{\xi }} }^{\langle n-m\rangle } (\varvec{\ddot{t}} \,)\right)$, $\widetilde{L}_{\varvec{\zeta },\varvec{\dot{\xi }}} (\varvec{\dot{t}}\,) =\phi _m\left( \mathbb {B}_{\varvec{\dot{\xi }}}^{\langle m\rangle } (\varvec{\dot{t}}\,)\right)$. Notice that $\psi _{n-m}\left( \mathbb {B}_{\varvec{\ddot{\xi }} }^{\langle n-m\rangle } (\varvec{\ddot{t}} \,)\right)$ is a symmetric function of $t^s_{1},\ldots , t_{\xi _s}^s$ for each $s=m+1,\ldots ,n-1$, while $\phi _m\left( \mathbb {B}_{\varvec{\dot{\xi }}}^{\langle m\rangle } (\varvec{\dot{t}}\,)\right)$ is a symmetric function of $t^p_{1},\ldots , t_{\xi _p}^p$ for each $p=1,\ldots ,m-1$. Therefore, formula (5.7) for $\xi _m=0$ reduces to formula (3.9),

$$\begin{aligned} \mathbb {B}_{\varvec{\xi }}(\varvec{t})v\,=\, \phi _m \left( \mathbb {B}_{\varvec{\dot{\xi }} }^{\langle m\rangle }(\dot{\varvec{t}})\right) \psi _m\left( \mathbb {B}_ {\varvec{\ddot{\xi }}}^{\langle n-m\rangle }(\ddot{\varvec{t}})\right) v. \end{aligned}$$

### Remark

For $m=1$ after a minor simplification, formula (5.7) reads

$$\begin{aligned}  &   \mathbb {B}_{\varvec{\xi }}(\varvec{t})v\,=\,\prod _{l=1}^{\xi _1}\,\dfrac{1}{t^{1}_l-x}\; \sum _{\varvec{q}}\;\prod _{i=2}^n\,\dfrac{e^{q_i}_{i1}}{q_i!}\,\, \prod _{b=2}^{n-1}\,\frac{1}{\left( \xi _b-\eta _b\right) !}\;{{\operatorname {Sym}}_{{\varvec{t}}^1}}\,{\operatorname {Sym}}_{\ddot{\varvec{t}}}\\    &   \left[ L_{\varvec{\eta },\varvec{\ddot{\xi }}}(\varvec{\ddot{t}})\,U_{\varvec{I}}\bigl (\varvec{\ddot{t}}_{[\varvec{\eta }]}, \varvec{t}^1\bigr )\Phi (\varvec{t}^1)\right] v\,, \end{aligned}$$

where the sum is over $\varvec{q}=(q_2,\ldots ,q_n)\in \mathbb Z_{\geqslant 0}^{n-1}$ such that $q_2+\dots +q_n=\xi _1$ and $\eta _b=q_{b+1}+\cdots +q_n\leqslant \xi _b$ for all $b=2,\ldots ,n-1$, while $\varvec{I}=(I_2,\ldots , I_n)$ is any partition of $\{1,\ldots ,\xi _1\}$ such that $|I_j|=q_j$ for all $j=2,\ldots ,n$. Thus for $m=1$, Theorem 5.5 becomes Theorem 3.3 in [23].

Similarly, for $m=n-1$ after a minor simplification, formula (5.7) reads

$$\begin{aligned}\begin{aligned} \mathbb {B}_{\varvec{\xi }}(\varvec{t})v\,= \,\,&\!\prod _{l=1}^{\xi _{n-1}}\dfrac{1}{t_{l}^{n-1}-x} \;\sum _{\varvec{q}}\;\prod _{j=1}^{n-1}\,\dfrac{e^{q_j}_{nj}}{q_j!}\,\, \prod _{a=1}^{n-2}\,\frac{1}{\left( \xi _a-\zeta _a\right) !} \\& \times {\operatorname {Sym}}_{{\varvec{t}}^{n-1}}\,{{\operatorname {Sym}}_{\dot{\varvec{t}}}}\, \left[ \widetilde{L}_{\varvec{\zeta },\varvec{\dot{\xi }}} (\varvec{\dot{t}}\,)\,\widetilde{U}_{\varvec{J}}\bigl (\varvec{\dot{t}}_{(\varvec{\dot{\xi }}-\varvec{\zeta },\varvec{\dot{\xi }}]},{\varvec{t}}^{n-1}\bigr )\,\Phi (\varvec{t}^{n-1})\right] v\,, \end{aligned}\end{aligned}$$

where the sum is over $\varvec{q}=(q_1,\ldots ,q_{n-1})\in \mathbb Z_{\geqslant 0}^{n-1}$ such that $q_1+\dots +q_{n-1}=\xi _{n-1}$ and $\zeta _a=q_1+\dots +q_a\leqslant \xi _a$ for all $a=1,\ldots ,n-2$, while $\varvec{J}=(J_1,\ldots , J_{n-1})$ is any partition of $\{1,\ldots ,\xi _{n-1}\}$ such that $|J_i|=q_i$ for all $i=1,\ldots ,n-1$. Thus for $m=n-1$, Theorem 5.5 becomes Theorem 3.1 in [23].

## Proof of Theorem 5.5

Throughout this section, we use the notation in Theorem 5.5. We will begin with two auxiliary lemmas.

### Lemma 6.1

Consider functions $\,F(x_1,\ldots ,x_{k})$ and $\,G(x_1,\ldots ,x_l)$, $\,k\leqslant l$, such that $\,G(x_1,\ldots ,x_l)$ is a symmetric function of $x_1,\ldots ,x_{k}$ and of $x_{k+1},\ldots ,x_l$. Then,

$$\begin{aligned} \begin{aligned} {\operatorname {Sym}}_{x_1,\ldots ,x_l}&\;\bigl [F(x_1,\ldots ,x_{k}) G(x_1,\ldots ,x_l)\bigr ]\\&\quad =\dfrac{1}{k!}\,{\operatorname {Sym}}_{x_1,\ldots ,x_k} \;\Bigl [\Bigl ({\operatorname {Sym}}_{x_1,\ldots ,x_{l} }F(x_1,\ldots ,x_{k}) \Bigr )G(x_1,\ldots ,x_l)\Bigr ]. \end{aligned}\end{aligned}$$

### Proof

By inspection. $$\square $$

Recall the weight functions $W_{\varvec{I}}(\varvec{v}; \varvec{z})$, $\widetilde{W}_{\varvec{J}}(\varvec{u}; \varvec{z})$, see (4.3),(4.4), and the functions $L_{\varvec{\eta },\varvec{\ddot{\xi }}}(\varvec{\ddot{t}},\varvec{t}^m )$, $\widetilde{L}_{\varvec{\zeta },\varvec{\dot{\xi }}} (\varvec{\dot{t}},\varvec{t}^m )$ given by (5.1),(5.2).

### Lemma 6.2

One has

$$\begin{aligned} {\operatorname {Sym}}_{\varvec{\ddot{t}}}\left[ U_{\varvec{I}}\left( \varvec{\ddot{t}}_{[\varvec{\eta }]}, \varvec{t}^m \right) L_{\varvec{\eta },\varvec{\ddot{\xi }}}(\varvec{\ddot{t}}\,) \right] =\prod _{s=m+1}^{n-1} \dfrac{1}{\eta _s!}\;{\operatorname {Sym}}_{\varvec{\ddot{t}}} \left[ W_{\varvec{I}}\left( \varvec{\ddot{t}}_{[\varvec{\eta }]}, \varvec{t}^m \right) L_{\varvec{\eta },\varvec{\ddot{\xi }}}(\varvec{\ddot{t}}\,) \right] , \end{aligned}$$

$$\begin{aligned} {{\operatorname {Sym}}_{\varvec{\dot{t}}}} \left[ \widetilde{U}_{\varvec{J} }(\varvec{\dot{t}}_{(\varvec{\dot{\xi }}-\varvec{\zeta },\varvec{\dot{\xi }}]},\check{\varvec{t}}^m ) \widetilde{L}_{\varvec{\zeta },\varvec{\dot{\xi }}} (\varvec{\dot{t}}\,) \right] =\prod _{s=1}^{m-1} \dfrac{1}{\zeta _s!} \;{{\operatorname {Sym}}_{\varvec{\dot{t}}}} \left[ \widetilde{W}_{\varvec{J} }(\varvec{\dot{t}}_{(\varvec{\dot{\xi }}-\varvec{\zeta },\varvec{\dot{\xi }}]},\check{\varvec{t}}^m ) \widetilde{L}_{\varvec{\zeta },\varvec{\dot{\xi }}} (\varvec{\dot{t}}\,)\right] . \end{aligned}$$

### Proof

Observe that for every $s=m+1,\ldots ,n-1$, the function $L_{\varvec{\eta },\varvec{\ddot{\xi }}}(\varvec{\ddot{t}}\,)$ is symmetric in $t^s_{1},\ldots , t_{\eta _s}^s$ and in $t_{\eta _s+1}^s,\ldots , t^s_{\xi _s}$. Similarly, for every $s=1,\ldots ,m-1$, the function $\widetilde{L}_{\varvec{\zeta },\varvec{\dot{\xi }}} (\varvec{\dot{t}}\,)$ is a symmetric in $t^s_{1},\ldots , t_{\xi _s-\zeta _s}^s$ and in $t_{\xi _s-\zeta _s+1}^s,\ldots , t^s_{\xi _s}$. Then, the statement follows from the formulas (4.3),(4.4) by applying Lemma 6.1 several times. $\square$

Recall that for $\sigma \in S_{\xi _m}$ and a partition $\varvec{J}=\left( J_1, \ldots , J_m\right)$ of $\{1,\ldots ,\xi _m\}$, we have $\sigma (\varvec{J})=\left( \sigma \left( J_1\right) , \ldots , \sigma \left( J_m\right) \right)$. Similarly, for $\varvec{I}=(I_{m+1},\ldots ,I_n)$, we have $\sigma (\varvec{I})=\left( \sigma \left( I_{m+1}\right) , \ldots , \sigma \left( I_{n}\right) \right)$.

The next proposition is the key point of the proof. For a collection $\varvec{z}=(z_1,\ldots , z_{\xi _m})$, set

$$\begin{aligned} \Phi (\varvec{z})= \prod _{1\leqslant a<b\leqslant \xi _m} \dfrac{z_a-z_b-1}{z_a-z_b} . \end{aligned}$$

### Proposition 6.3

One has

6.1 $$\begin{aligned} \begin{aligned} \sum _{\sigma \in S_{\xi _m}}W_{\sigma (\varvec{I})}\left( \varvec{\ddot{t}}_{[\varvec{\eta }]}, \varvec{z} \right)&\widetilde{W}_{\sigma (\varvec{J})}(\varvec{\dot{t}}_{(\varvec{\dot{\xi }}-\varvec{\zeta },\varvec{\dot{\xi }}]},\varvec{z} ) \\&={ {\operatorname {Sym}}_{\varvec{z} }}\Bigl [W_{\sigma _0(\varvec{I})}(\varvec{\ddot{t}}_{[\varvec{\eta }]}, \varvec{z})\widetilde{W}_{\varvec{J}}(\varvec{\dot{t}}_{(\varvec{\dot{\xi }}-\varvec{\zeta },\varvec{\dot{\xi }}]}, {\varvec{z}}^{\sigma _0}) \Phi (\varvec{z})\Bigr ] , \end{aligned}\end{aligned}$$

where $\sigma _0$ is the longest permutation in $S_{\xi _m}$, $\sigma _0(i)=\xi _m-i+1$, and $\varvec{z}^{\sigma _0}=(z_{\xi _m},\ldots ,z_1)$.

### Proof

Formula (6.1) can be written as:

6.2 $$\begin{aligned}  &   \sum _{\sigma \in S_{\xi _m}}W_{\sigma (\varvec{I})}\left( \varvec{\ddot{t}}_{[\varvec{\eta }]}, \varvec{z} \right) \widetilde{W}_{\sigma (\varvec{J})}(\varvec{\dot{t}}_{(\varvec{\dot{\xi }}-\varvec{\zeta },\varvec{\dot{\xi }}]},\varvec{z} ) \nonumber \\  &   =\sum _{\pi \in S_{\xi _m}} W_{\sigma _0(\varvec{I})}(\varvec{\ddot{t}}_{[\varvec{\eta }]}, \varvec{z}^\pi )\widetilde{W}_{\varvec{J}}(\varvec{\dot{t}}_{(\varvec{\dot{\xi }}-\varvec{\zeta },\varvec{\dot{\xi }}]}, {\varvec{z}}^{\pi \sigma _0}) \Phi (\varvec{z}^\pi ) , \end{aligned}$$

$\varvec{z}^\pi =(z_{\pi (1)},\ldots ,z_{\pi (\xi _m)})$. Then, the proof of formula (6.2) is literately the same as that of formula (7.16) in [6] with Corollary 4.1 in this paper substituting Lemma 7.5 in [6].$\square$

### Proof of Theorem 5.5:

First we apply Lemma 6.2 to formula (5.6) and get

6.3 $$\begin{aligned} \begin{aligned} \mathbb {B}_{\varvec{\xi }}(\varvec{t})v\,=\prod _{l=1}^{\xi _m}\,\dfrac{1}{t_{l}^m\!-\!x} \sum _{\varvec{q}\!\in \! \mathcal {Q}_{m,n}}\,\prod _{i\!=\!m\!+\!1}^n\,\prod _{j\!=\!1}^m\, e^{q_{ij}}_{ij}\, \prod _{a\!=\!1}^{m\!-\!1}\,\frac{1}{\left( \xi _a\!-\!\zeta _a\right) !\,\zeta _a!}\,\prod _{b\!=\!m\!+\!1}^{n\!-\!1} \frac{1}{\left( \xi _b\!-\!\eta _b\right) !\,\eta _b!}\!\!&\\  \times {\operatorname {Sym}}_{\varvec{\ddot{t}}} \;\! {{\operatorname {Sym}_{\varvec{\dot{t}}}}}\Biggl [ L_{\varvec{\eta },\varvec{\ddot{\xi }}}(\varvec{\ddot{t}}\,) \widetilde{L}_{\varvec{\zeta },\!\varvec{\dot{\xi }}} (\varvec{\dot{t}}\,)\!\sum _{(\varvec{I},\varvec{J})\! \in \! \mathcal {S}_{\varvec{q}}}\!\widetilde{W}_{\varvec{J}}(\varvec{\dot{t}}_{(\varvec{\dot{\xi }}\!-\!\varvec{\zeta },\varvec{\dot{\xi }}]},\varvec{t}^m ) W_{\varvec{I} }\left( \varvec{\ddot{t}}_{[\varvec{\eta }]}, \varvec{t}^m \right) \Biggr ]v\,.\!\!\!&\end{aligned}\end{aligned}$$

Notice that every pair $(\varvec{I},\varvec{J}) \in \mathcal {S}_{\varvec{q}}$ can be obtained from an arbitrary fixed pair $(\varvec{I}_0,\varvec{J}_0) \in \mathcal {S}_{\varvec{q}}$ by the action of the symmetric group $S_{\xi _m}$. Therefore, the inner sum in the right-hand side of formula (6.3) can be written in the following way,

6.4 $$\begin{aligned} \begin{aligned}&\hspace{-8.00003pt}\sum _{(\varvec{I},\varvec{J}) \in \mathcal {S}_{\varvec{q}}}W_{\varvec{I}}\left( \varvec{\ddot{t}}_{[\varvec{\eta }]}, \varvec{t}^m\right) \widetilde{W}_{\varvec{J}}(\varvec{\dot{t}}_{(\varvec{\dot{\xi }}-\varvec{\zeta },\varvec{\dot{\xi }}]},\varvec{t}^m)\\& \quad =\prod _{i=m+1}^n\,\prod _{j=1}^m\,\frac{1}{q_{ij}!}\,\sum _{\sigma \in S_{\xi _m}} W_{\sigma (\varvec{I_0})}\left( \varvec{\ddot{t}}_{[\varvec{\eta }]}, \varvec{t}^m\right) \widetilde{W}_{\sigma (\varvec{J_0})}(\varvec{\dot{t}}_{(\varvec{\dot{\xi }}-\varvec{\zeta },\varvec{\dot{\xi }}]},\varvec{t}^m)\,. \end{aligned}\end{aligned}$$

Here $\prod _{i,j} {(q_{i,j})!}$ is the cardinality of the isotropic subgroup of the pair $(\varvec{I_0},\varvec{J_0})$. Applying Proposition 6.3 for $\varvec{z}=\varvec{t}^{m}$ to the sum over permutations $\,\sum _{\sigma \in S_{\xi _m}}\!$ in the right-hand side of (6.4), we get

$$\begin{aligned}\begin{aligned} \mathbb {B}_{\varvec{\xi }}(\varvec{t})v\,= \,&\prod _{l\!=\!1}^{\xi _m}\dfrac{1}{t_{l}^m\!-\!x} \sum _{\varvec{q}\!\in \!\mathcal {Q}_{m,n}}\,\prod _{i\!=\!m\!+\!1}^n\,\prod _{j\!=\!1}^m\,\dfrac{e^{q_{ij}}_{ij}}{q_{ij}!}\,\, \prod _{a\!=\!1}^{m\!-\!1}\,\frac{1}{\left( \xi _a\!-\!\zeta _a\right) !\,\zeta _a!}\,\prod _{b\!=\!m\!+\!1}^{n\!-\!1} \frac{1}{\left( \xi _b\!-\!\eta _b\right) !\,\eta _b!} \\& \times {{\operatorname {Sym}}_{{\varvec{t}}^m}} \,\!{{\operatorname {Sym}}_{\dot{\varvec{t}}}}\!\,{\operatorname {Sym}}_{\ddot{\varvec{t}}} \Bigg [ L_{\varvec{\eta },\!\varvec{\ddot{\xi }}}(\varvec{\ddot{t}}\,) \widetilde{L}_{\varvec{\zeta },\!\varvec{\dot{\xi }}} (\varvec{\dot{t}}\,) \; \!\widetilde{W}_{\varvec{J}_0}(\varvec{\dot{t}}_{(\varvec{\dot{\xi }}\!-\!\varvec{\zeta },\!\varvec{\dot{\xi }}]},\check{\varvec{t}}^m )\\  &W_{\varvec{\check{I}}_0}\!\left( \varvec{\ddot{t}}_{[\varvec{\eta }]}, \varvec{t}^m \right) \Phi (\varvec{t}^m) \Bigg ]v\,. \end{aligned}\end{aligned}$$

Finally, we use Lemma 6.2 once more to transform the symmetrization ${{\operatorname {Sym}}_{\dot{\varvec{t}}}}\,{\operatorname {Sym}}_{\ddot{\varvec{t}}}$ in the last formula to the form in formula (5.7). $\square$

## Data Availability

Additionally, data sharing is not applicable to this article, as no datasets were generated or analyzed during the current study.
